# Association of residential neighborhood disadvantage with amyloid PET positivity among cognitively impaired individuals

**DOI:** 10.1002/bsa3.70058

**Published:** 2026-01-29

**Authors:** Charles C. Windon, Elena Tsoy, Jennifer Livaudais-Toman, Torsten B. Neilands, Nynikka R. Palmer, Margo B. Heston, Julene K. Johnson, Lucy Hanna, Constantine Gatsonis, Justin Romanoff, Jon Steingrimsson, Maria C. Carrillo, Peggye Dilworth-Anderson, Bruce E. Hillner, Barry A. Siegel, Rachel A. Whitmer, Consuelo H. Wilkins, Karen Smith, Ganna Blazhenets, Renaud La Joie, Bruce L. Miller, Katherine L. Possin, Gil D. Rabinovici

**Affiliations:** 1 Department of Neurology, Memory and Aging Center, Weill Institute for Neurosciences, University of California, San Francisco, San Francisco, California, USA; 2 Global Brain Health Institute (GBHI), University of California, San Francisco, San Francisco, California, USA; 3 Department of Medicine, Division of General Internal Medicine, University of California, San Francisco, San Francisco, California, USA; 4 Center for Aging in Diverse Communities, University of California, San Francisco, San Francisco, California, USA; 5 University of California San Francisco School of Medicine, San Francisco, California, USA; 6 Department of Medicine, Division of General Internal Medicine, Zuckerberg San Francisco General Hospital, University of California San Francisco, San Francisco, California, USA; 7 University of California San Francisco Institute for Health & Aging, San Francisco, California, USA; 8 Center for Statistical Sciences, Brown University School of Public Health, Providence, Rhode Island, USA; 9 Department of Biostatistics, Brown University School of Public Health, Providence, Rhode Island, USA; 10 Alzheimer’s Association, Chicago, Illinois, USA; 11 Health Policy and Management, Gillings School of Global Public Health, University of North Carolina at Chapel Hill, Chapel Hill, North Carolina, USA; 12 Department of Medicine, Virginia Commonwealth University, Richmond, Virginia, USA; 13 Edward Mallinckrodt Institute of Radiology, Washington University School of Medicine, St. Louis, Missouri, USA; 14 Division of Research, Kaiser Permanente, Pleasanton, California, USA; 15 Department of Public Health Sciences, University of California, Davis, Davis, California, USA; 16 Department of Medicine, Division of Geriatric Medicine, Vanderbilt University Medical Center, Nashville, Tennessee, USA; 17 Department of Radiology and Biomedical Imaging, University of California, San Francisco, San Francisco, California, USA

**Keywords:** Alzheimer’s disease, amyloid positron emission tomography, dementia, ethnicity, Medicare, mild cognitive impairment

## Abstract

**INTRODUCTION:**

Relationships between Alzheimer’s disease neuropathology, residential neighborhood, and cognitive impairment remain incompletely understood.

**METHODS:**

We examined whether residence within a disadvantaged neighborhood was associated with amyloid positron emission tomography (PET) positivity. We used data from the observational, multisite, Imaging Dementia–Evidence for Amyloid Scanning study that included cognitively impaired Medicare beneficiaries. Our secondary analysis examined multivariable-adjusted associations between neighborhood disadvantage (measured by Area Deprivation Index [ADI] deciles 1–90 vs. 91–100 representing greatest disadvantage) and amyloid PET positivity.

**RESULTS:**

Among 15,346 White, 829 Latino, 637 Black/African American, and 321 Asian individuals, 51% were female, mean age was 75.7 years, 535 (3.8%) resided in ADI 91 to 100 decile, and 61.6% were amyloid PET positive. The ADI 91–100 decile was associated with lower odds of PET positivity by visual interpretation (odds ratio [OR] 0.80, 95% confidence interval [CI] 0.67–0.96, *p* ≤ .001) but not PET Centiloid value ≥ 40 versus ≤ 10 (OR 0.81, 95% CI 0.66–1.01, *p* = 0.060).

**CONCLUSION:**

Residence in the most disadvantaged neighborhoods may be associated with lower amyloid pathology in cognitively impaired individuals.

## 1 | INTRODUCTION

Characterizing Alzheimer’s disease (AD) biomarkers in vivo is critical in correctly identifying underlying neuropathology. These biomarkers are increasingly important when evaluating an individual for symptoms of cognitive impairment, given the numerous potential causes of cognitive impairment. Amyloid positron emission tomography (PET) is a gold-standard tool for detecting amyloid pathology and is increasingly used clinically in the United States due to greater coverage for Medicare patients since June 2023. PET can be interpreted visually (as positive, suggesting an elevated brain accumulation of amyloid beta [A*β*], or negative) or quantitatively by applying a scale of “Centiloid” (CL) units.^1^

Disadvantaged neighborhoods are notable for poverty, high unemployment rates, and lack of access to healthy foods, among other characteristics.^2–3^ Residents of these neighborhoods may experience poorer health outcomes overall compared to their counterparts living in less disadvantaged settings.^4^ Neighborhood disadvantage has also been associated with greater risk of clinical dementia^5^ and AD neuropathology.^6^ *Post mortem* studies of 447 decedents from two Alzheimer’s Disease Research Centers revealed each decile increase in neighborhood disadvantage (as measured by the Area Deprivation Index [ADI]) was associated with 8.1% increased odds of AD neuropathology at autopsy.^6^ ADI is a multicomponent measure of a region’s socioeconomic conditions that includes core domains of income, education, employment, and housing quality.^7^

Additional work examining decedents in the UK Brains for Dementia Research Initiative revealed residents from the 20% most disadvantaged neighborhoods had significantly higher AD-specific neuropathology at autopsy: neurofibrillary tangles and neuritic plaques as well as cerebral amyloid angiopathy.^8^ The mechanism by which residence in a disadvantaged neighborhood, as characterized by ADI or other measures, impacts AD neuropathology and health can be understood through multiple frameworks. For example, the previously proposed social ecological model and subsequent work^9–10^ provide a means of understanding how factors like education, income, housing, and employment (core ADI domains) significantly influence health and rates of disease.

Ethnoracial differences in in vivo biomarkers of amyloid and other AD neuropathology have been described but multiple questions remain regarding these described differences given inconsistent conclusions across studies.^11–17^ Clinicopathological studies have also revealed mixed findings, specifically inconsistent findings regarding amyloid plaque and neurofibrillary tangle burden in Latino compared to non-Latino White individuals, and similar inconsistencies comparing Black/African American and non-Latino White individuals.^18^ Research regarding additional ethnoracial groups is scarce. Of note, prior biomarker and clinicopathological studies have often been relegated to single sites and many lack broad geographical and sociodemographic diversity of participants, which limits the conclusions that can be drawn from them.

It remains unknown if residing in a disadvantaged neighborhood is associated with in vivo PET measurement of amyloid pathology, particularly among ethnoracially diverse individuals in the United States, many of whom live in more disadvantaged neighborhoods due to discriminatory housing practices and other factors.^19–20^ Understanding rates of AD and non-AD pathology among individuals across the spectrum of privilege and disadvantage may help to better characterize burden of disease across communities. This study’s primary aim was to examine whether neighborhood disadvantage, as measured by ADI,^21^ is associated with amyloid PET positivity, as defined by visual PET interpretation. We leveraged data from the Imaging Dementia–Evidence for Amyloid Scanning (IDEAS) study, a US nationally representative study that evaluated the clinical utility of amyloid PET in Medicare beneficiaries over age 65 who met criteria for mild cognitive impairment (MCI) or dementia.^22^ We hypothesized that higher ADI would be associated with higher rates of amyloid PET positivity by visual read across ethnoracial groups. For our secondary objective, we examined whether neighborhood disadvantage was associated with amyloid PET positivity quantified by CL value.^1^ We again hypothesized that higher ADI would be associated with higher rates of amyloid PET positivity, defined by CL ≥ 40 (quantitatively positive scan) versus CL ≤ 10 (quantitatively negative scan). Across the aims, we explored whether living in high neighborhood disadvantage had a differential effect on rates of amyloid PET positivity across ethnoracial groups.

## 2 | MATERIALS & METHODS

### 2.1 | Study population

The IDEAS Study included 18,293 cognitively impaired Medicare beneficiaries receiving clinical care from dementia specialists across the United States between 2016 and 2018.^22^ IDEAS examined the impact of amyloid PET on clinical management and 12 month health outcomes among individuals meeting appropriate use criteria for amyloid PET.^23^ Five-hundred ninety-five dementia practices and 343 PET imaging facilities enrolled participants into the IDEAS study. Additional study details have been published elsewhere.^22,24^ Data from visual interpretation of amyloid PET scans were available for 18,256/18,293 (99%) patients, and CL values were available for 10,361/18,293 (57%) patients (Figure S1 in supporting information). CL values were not calculated during the IDEAS study itself (not a primary goal of the study) but rather calculated after study completion for a subset of individuals for whom CL calculation was possible based on imaging data availability.^25^ In this secondary analysis of IDEAS data, we included participants’ amyloid PET data, residential neighborhood disadvantage measures (available for 14,812/18,293, 81% of participants; Figure S1), and demographic and clinical data.

### 2.2 | Informed consent

The current study was approved by the University of California San Francisco Institutional Review Board (IRB; #23–39894), and the IDEAS study was approved by a central IRB (Advarra). Prior to participant enrollment, all participants provided informed consent. Participants also had the option to consent via legal representative if they lacked capacity to consent.^22^

### 2.3 | Race, ethnicity, and sex classification

At study enrollment in IDEAS, dementia specialists captured racial identity using the following categories: American Indian or Alaska Native, Asian, Black or African American, Native Hawaiian or Pacific Islander, White, other, and prefer not to answer. Ethnicity was captured as: Hispanic or Latino, not Hispanic or Latino, other, or prefer not to answer.^26^ There were no specific instructions provided to dementia specialists on how to collect information about race and ethnicity in IDEAS, though all data was self-reported from patients. For our current analysis, individuals who identified as Hispanic or Latino were classified as Latino, regardless of identified race. All other individuals were classified by racial identity. Individuals who identified as American Indian or Alaska Native, Native Hawaiian or Pacific Islander, more than one race, other, or prefer not to answer were not included in analyses due to small group sizes. Our final categories for analysis included non-Latino White, Latino, Black/African American, and Asian.

Participants provided their self-identified sex in IDEAS from the following options: male, female, transgender male, transgender female, none of these fully describe me (allow free text), prefer not to answer.^26^ No specific instructions were provided to dementia specialists on how to collect this information.

### 2.4 | PET acquisition and interpretation in IDEAS

Amyloid PET was acquired in IDEAS for 18,256 patients using one of three US Food and Drug Administration–approved tracers^23^ across the 343 participating PET imaging facilities. Radiologists and nuclear medicine specialists at IDEAS imaging sites performed visual PET interpretation according to published tracer-specific instructions.^27–29^ Radiologists at each site classifieds scans as positive (indicating tracer retention within cortical regions, suggestive of elevated brain accumulation of A*β*) or negative (tracer isolated retention confined to white matter only).

### 2.5 | CL value calculation for amyloid PET scans

The amyloid PET images of 10,361 IDEAS participants in DICOM (Digital Imaging and Communications in Medicine) format were converted to NifTI (Neuroimaging Informatics Technology Initiative) format via dicm2nii^30^ and processed with an in-house PET-only processing pipeline (rPOP).^31^ rPOP is a MATLAB-based and magnetic resonance imaging (MRI)-free (does not require structural MRI) pipeline process that warps images to template space and smooths them to a common resolution, then extracts cortical (target region) and cerebellar (reference region) counts to calculate standardized uptake value ratios (SUVR). rPOP then converts SUVR to CL units using validated transformation equations.^1^ CL range from < 0 to > 100, with 0 representing mean tracer uptake in healthy young adults who lack AD neuropathology and 100 representing mean tracer uptake in patients with mild–moderate dementia due to AD.^32^ Based upon previously validated cutoffs, we defined amyloid PET positivity as CL ≥ 40 and amyloid PET negativity as CL ≤ 10, with CL 11 to 39 representing an “intermediate zone.”^25,32–36^

### 2.6 | Data geocoding and ADI generation

Neighborhood disadvantage was measured with the national-level ADI, a multicomponent composite measure of a region’s socioeconomic conditions that is validated at the Census block group level.^21^ ADI measures were available for 14,812/18,293 study participants and provided directly from the IDEAS study team. ADI includes the core domains of income, education, employment, and housing quality across 17 US Census indicators as described elsewhere.^7^ The IDEAS study team used a C# programming language and leveraged a multi-tiered approach to extract valid national-level ADI data from the Neighborhood Atlas^21^ for each IDEAS participant. The program first attempted to validate the full participant residential address (collected for each IDEAS participant) using the US Postal Service address validation application programming interface. If a valid address was found, the US Census Bureau’s official geocoder was used to identify a geocode corresponding to the address using 2020 Census block data. If the address was not validated, the individual’s ZIP code+4 digits was used to search for the geocode. If still no geocode was identified, a 5-digit ZIP code was used to find the geocode. The IDEAS study team then used the geocodes to look up the ADI scores from the Neighborhood Atlas, University of Wisconsin School of Medicine and Public Health (2022, Version 4). If these sources were unable to determine the geocodes, the participant’s ADI scores were set to “missing.”

The most severe disparities in AD neuropathology outcomes have been observed among individuals living in areas with the highest disadvantage.^6,8^ This guided our decision to dichotomize ADI score into 1 to 90 versus 91 to 100, using prior research methodology^7,37^ (values of 1–90 representing neighborhoods of lower disadvantage and values of 91–100 representing neighborhoods of greatest disadvantage). We also performed analyses examining ADI across deciles as a categorical variable and compared findings to results from dichotomization of ADI.

### 2.7 | Statistical analysis

Baseline participant demographics were summarized first by ethnoracial identity (non-Latino White, Latino, Black/African American, Asian) and then by amyloid PET positivity status (positive vs. negative by visual interpretation). Summaries included mean and standard deviation (SD) for continuous variables and count and percentage per stratum of categorical variables. Chi-squared analysis (accounting for clustering of patients within practices using the svy commands in Stata) was performed to examine differences in patient characteristics across ethnoracial groups. Given that IDEAS participants were nested within clinical practices, we used a multilevel binary logistic random intercepts model to account for the correlation between observations from patients in the same practice and to describe the relationship between ADI and binary visual interpretation of amyloid PET positivity.

To test whether higher ADI was related to higher rates of quantitative amyloid PET positive (CL ≥ 40) or indeterminate (CL 11–39) status, we used a multilevel multinomial logistic mixed effects model with negative status (CL ≤ 10) as the reference outcome and a random intercept for clinical practice. All models adjusted for age (continuous years, not centered), sex (binary, male reference), ethnoracial identity (categorical, non-Latino White reference), education (binary, high school or less reference), primary language (categorical, English reference), medical comorbidities (binary for each medical condition, no history of the condition as reference), impairment level (binary, mild cognitive impairment, reference), and Mini-Mental State Examination (MMSE, continuous; patients with a Montreal Cognitive Assessment score [MoCA] and missing MMSE score [*N* = 3929, 20% of participants] had MoCA score converted to MMSE using published methods^38^). Medical comorbidities included hypertension, other cardiovascular conditions (congestive heart failure, atrial fibrillation, history of acute or myocardial infarction, ischemic heart disease, dyslipidemia), pulmonary conditions (chronic obstructive pulmonary disease [COPD]), diabetes, chronic kidney disease, mood disorders (depression, bipolar affective disorder, schizophrenia), and cerebrovascular disease (cerebrovascular disease without stroke, prior history of stroke or transient ischemic attack).

We used Pearson correlation to examine correlations between seven medical comorbidities (hypertension, other cardiovascular disease, pulmonary disease, diabetes, chronic kidney disease, mood disorder, cerebrovascular disease). Several of the comorbidities were significantly correlated with one another (*p* < 0.001), but the absolute values of all correlations were < 0.36, and thus all comorbidities were included in the models described above.

We examined whether ADI effects on amyloid positivity rates were moderated by ethnoracial identity by adding interaction terms into the model (categorical, ADI 1–90, non-Latino White reference), age (continuous, ADI × age), and sex (categorical, ADI 1–90, male reference), and excluded non-significant interaction terms from the final models. Across models we applied multiple imputation (using M*plus*) with an unrestricted random effects model to generate 100 imputed data sets, per best practices,^39^ that included imputed values for missing covariates, ADI, and/or amyloid positivity values. For the model of CL-based positivity as the outcome, we included amyloid PET visual interpretation (positive vs. negative) as an auxiliary variable for the imputation due to high concordance between CL and visual interpretation. No adjustments for multiplicity were made. All statistical analyses were performed using Stata version 16.1 and M*plus* Version 8.10.

Given varying amounts of missingness of ADI data and CL value data, we also ran a generalized estimating equation model with exchangeable correlation (listwise deletion model) to estimate the association of ADI and visual amyloid PET positivity using data from individuals only with non-missing ADI data. Additionally, we ran a nominal logistic regression with cluster adjustment (listwise deletion model) to estimate the association of ADI with CL value using data from individuals only with non-missing CL value data.

## 3 | RESULTS

### 3.1 | Participant characteristics by ethnoracial group

Participant demographics by ethnoracial identity are reported in Table 1. This table includes 17,136 individuals with known ethnoracial identity and excludes 1157 with “other/prefer not to answer” identity. The mean age within the overall sample was 75.7 (SD = 6.3) years and Asian participants were the oldest (mean 76.3 years, SD 6.3, *p* = 0.031). There was greater female representation among Black/African American (62%) and Latino (62%) individuals (*p* < 0.001). Total years of education attainment was lowest among Latino individuals (62% with high school or less education, *p* < 0.001). Hypertension was most prevalent among Black/African American individuals (68%, *p* < 0.001) and diabetes was least prevalent among non-Latino White individuals (14%, *p* < 0.001). The highest dementia rates were observed for Latino individuals (55%) followed by Black/African American individuals (52%; *p* < 0.001).

More than 8% of Black/African American individuals lived in ADI 91 to 100 neighborhoods, which differed from all other groups (3.7% non-Latino White, 3.6% Latino, 2.7% Asian individuals, *p* < 0.001; Table 1). The US geographic distribution of the cohort showed that Atlantic and Southern regions had greater representation across the total sample. Compared to Central and Western-Pacific areas these regions also had both higher proportions of Black/African American and Latino participants (Table S1 in supporting information).

### 3.2 | Visual read amyloid positivity rates and ADI by ethnoracial group

Amyloid PET positivity rates among Black/African American (54%), Latino (54%), Asian (45%), and non-Latino White (62%) individuals differed (*p* < 0.001; Table 1).

### 3.3 | Participant characteristics by visual read amyloid PET positivity

Demographics and clinical characteristics are summarized by amyloid PET positivity (visual interpretation) in Table 2. This table includes data for a total of 18,256 individuals with non-missing data for amyloid PET. Those living in ADI 1 to 90 neighborhoods had significantly higher rates of positive amyloid PET compared to those living in ADI 91 to 100 neighborhoods (61.4% vs. 53.6%, *p* < 0.001). Females had higher rates of amyloid PET positivity compared to males (62.6% vs. 59.4%, *p* < 0.001). Individuals with hypertension (60.1%, *p* = 0.016), pulmonary disease (50.2%, *p* < 0.001), diabetes (51.6%, *p* < 0.001), kidney disease (54.8%, *p* = 0.006), mood disorders (55.8%, *p* < 0.001), and cerebrovascular disease (57%, *p* < 0.001) had significantly lower rates of PET positivity compared to those without the conditions, and compared to the overall sample (Table 2). Individuals with dementia had higher rates of PET positivity compared to those with MCI (69.7% vs. 55.3%, *p* < 0.001).

Rates of dementia among those living in ADI 91 to 100 neighborhoods were not significantly different from rates of dementia among those living in ADI 1 to 90 neighborhoods (36.1%, 95% confidence interval [CI]: 30.4%–41.7% vs. 38.7%, 35.9%–41.4%, respectively; *p* = 0.295; data not presented in tables). Rates of MCI were 61.3% (58.6%–64.1%) among individuals in ADI 1 to 90 neighborhoods and 63.9% (58.3%–69.6%) among individuals in ADI 91 to 100 neighborhoods, respectively.

### 3.4 | Multivariable model of ADI and PET positivity by visual read

Results of the binomial logistic mixed effects model of visual amyloid PET positivity status on ADI are presented in Table 3. This includes data for a total of 18,293 individuals, all missing values imputed. There were differences in demographics between individuals with non-missing ADI data compared to individuals with missing ADI data. Females, Black/African American individuals, those with high school education or less, those with hypertension, those with diabetes, and those with dementia were more likely than their counterparts to have missing ADI data (Table S2 in supporting information).

Individuals living in ADI 91 to 100 neighborhoods had lower odds (odds ratio [OR] 0.80, 95% CI 0.67–0.96, *p* = 0.017) of amyloid PET positivity, compared to those living in ADI 1 to 90 neighborhoods. Examination of ADI as a categorical variable (1–90) in a generalized estimating equation model with exchangeable correlation (listwise deletion model) also yielded nearly identical results (Table S3 in supporting information). Interactions between ADI and ethnoracial identity, ADI and age, and ADI and sex were not significant.

### 3.5 | Multivariable model of ADI and PET positivity by CL value

Results of the multinomial logistic regression examining ADI and amyloid PET positivity defined by CL value (≥ 40 positive; ≤ 10 negative; 11–39 “intermediate” zone) are displayed in Table 4. This includes data for a total of 18,293 individuals, all missing values imputed. There were minimal differences in demographics between individuals with CL value data compared to individuals missing CL value data. Those with hypertension, other vascular comorbidities, and those with dementia (vs. MCI) were slightly more likely than their counterparts to have missing CL value data (Table S4 in supporting information).

Compared to residents in ADI 1 to 90 neighborhoods, those in ADI 91 to 100 neighborhoods did not have lower odds of CL ≥ 40 versus CL ≤ 10 (OR 0.81, 95% CI 0.66–1.01, *p* = 0.060). The effect of ADI was also non-significant when evaluating odds of CL 11 to 39 versus CL ≤ 10 (OR 0.74, 0.53–1.02, *p* = 0.062). Examination of ADI as a categorical variable (1–90) in a nominal logistic regression with cluster adjustment (listwise deletion model) also revealed residents in ADI 91 to 100 neighborhoods did not have lower odds of CL ≥ 40 versus CL ≤ 10 and the effect of ADI was non-significant when evaluating odds of CL 11 to 39 versus CL ≤ 10 (Table S5 in supporting information). Interactions between ADI and ethnoracial identity, ADI and age, and ADI and sex were not significant.

## 4 | DISCUSSION

Our secondary analysis of data from the large, multi-site IDEAS study revealed that older adults with MCI or dementia who resided in the 10% most disadvantaged neighborhoods (ADI 91–100) had lower odds of visual amyloid PET positivity than those living in the 90% least disadvantaged neighborhoods (ADI 1–90). We also observed Black/African American, Latino, and Asian individuals had lower rates of PET positivity, compared to non-Latino White individuals. We did not, however, observe a statistically significant race-by-ADI interaction on rates of visual or quantitative amyloid positivity. This study represents the largest examination to date of amyloid PET positivity, ethnoracial identity, and level of residential neighborhood disadvantage across the United States. Our study is also the first to examine the relationship between amyloid PET CL and neighborhood disadvantage among a large sample of ethnoracially diverse individuals.

### 4.1 | Neighborhood disadvantage and amyloid PET positivity

Individuals residing within the most disadvantaged neighborhoods had lower odds of visual amyloid PET positivity. Effects with visual reads were not replicated in tests of quantitative amyloid PET positivity, however. Our results contrast with previously reported findings, which are mixed.

*Post mortem* examination of 447 decedents from two Alzheimer’s Disease Research Centers revealed that higher ADI was associated with increased odds of AD neuropathologic change at autopsy.^6^ Notably, this sample only included 24 individuals from the 20% most disadvantaged neighborhoods and had small total numbers of Black/African American, Latino, and Asian individuals compared to our sample. This study only included a geographic sampling of two metropolitan areas in the United States. The geographic, socioeconomic, and ethnoracial distributions of the IDEAS sample afforded greater ability to detect ADI associations with amyloid PET positivity but because the study did not collect autopsy data we are unable to test relationships with *post mortem* amyloid pathology burden and directly compare our findings to clinicopathological studies. Another examination of 469 decedents with varying cognitive impairment severity from a single academic center revealed that neighborhood disadvantage was not associated with AD neuropathologic change or cerebrovascular pathology,^40^ but cognitive function was associated with neighborhood disadvantage independent of changes in dementia-associated neuropathology. This study importantly had limited representation of individuals from the most disadvantaged neighborhoods (159 total individuals), and 416/469 participants were White (389/469 non-Latino). For additional consideration is an examination of 789 decedents from the UK with varying levels of cognitive impairment that revealed those residing in the 20% most disadvantaged neighborhoods had greater neurofibrillary tangle and neuritic plaque staging at autopsy.^8^ This study featured a larger total proportion of individuals residing in the most disadvantaged neighborhoods (≈ 9%) compared to our study (≈ 4%). The total number of individuals from the most-deprived neighborhoods in this study was 68 compared to 535 in our study. This study also did not include more comprehensive medical comorbidity data as our study did, and had a substantial number of cognitively normal individuals included. IDEAS only enrolled cognitively impaired participants; thus, we were unable to include cognitively normal individuals in our analyses.

We did not replicate findings when examining a quantitative measure of amyloid PET positivity (CL value). We hypothesize this is secondary to the CL value cutoff used to define positivity (≥ 40) in our study. As noted, more than a single validated cutoff exists based on prior literature^25,32–36^ and examination of other positivity cutoffs, particularly those that are < 40, is an important future direction. Additionally, CL values can underestimate amyloid pathology due to multiple factors (image processing, reference region uptake, etc.)^31^ even when a scan is clearly visually positive. These factors could have contributed to our findings.

CL range from < 0 to > 100, with 0 representing mean tracer uptake in healthy young adults who lack AD neuropathology and 100 representing mean tracer uptake in patients with mild–moderate dementia due to AD.^32^ Based upon previously validated cutoffs, we defined amyloid PET positivity as CL ≥ 40 and amyloid PET negativity as CL ≤ 10, with CL 11 to 39 representing an “intermediate zone.”^25,32–36^

Non-amyloid pathology including cerebrovascular disease may make important contributions to cognitive impairment among those living in disadvantaged neighborhoods. An examination of > 19,000 individuals from the UK Biobank revealed that residence in a less disadvantaged neighborhood was associated with lower levels of cerebrovascular pathology.^41^ Our study found lower rates of amyloid PET positivity among individuals with conditions like hypertension, diabetes, chronic kidney disease, and cerebrovascular disease (by clinical history). Vascular risk factors like hypertension and diabetes have been demonstrated to contribute to cerebrovascular disease.^42^ It is possible the lower rates of amyloid PET among these participants reflect cognitive impairment driven by non-amyloid pathology like cerebrovascular disease, though lack of direct measures of cerebrovascular disease (and non-AD pathology) in IDEAS limits the conclusions we can draw regarding non-amyloid pathological contributions in our study. Future work should continue to explore this.

Considered in the context of the social ecological model, the mechanism by which residence in a disadvantaged neighborhood could lead to greater cerebrovascular disease or non-AD pathology is unclear. Prior work examining 740 decedents from two Alzheimer’s Disease Research Centers found greater number of years of residence in more disadvantaged neighborhoods (as measured by ADI) led to greater burden of cerebrovascular disease among individuals.^43^ Other work has revealed elevated cerebrospinal fluid inflammatory biomarkers among residents of more disadvantaged neighborhoods.^44^ Whether residence in a disadvantaged neighborhood creates a pro-inflammatory and altered environment within the brain that preferentially favors cerebrovascular disease and non-amyloid pathology development is not directly answerable within the context of our study but possibly an area for future work.

Survivorship bias and selection bias are important considerations in interpreting the results of our study. IDEAS only enrolled cognitively impaired individuals; thus, individuals from highly disadvantaged neighborhoods with high levels of amyloid pathology could have been excluded if cognitively normal. Numerous disparities have also been described in timeliness and accuracy of diagnosis of cognitive impairment. These disparities disproportionately affect minority communities and potentially individuals residing in highly disadvantaged communities.^45–46^ Individuals with high amyloid burden from highly disadvantaged neighborhoods also might not have enrolled in IDEAS due to greater disability and mortality rates, which could have impacted ability to participate. For example, our finding of higher rates of medical comorbidities among amyloid PET–negative individuals could be the result of premature mortality of individuals with these same medical comorbidities but also concurrent amyloid PET positivity.

### 4.2 | Ethnoracial identity, clinical profiles, PET positivity, and ADI

We observed prior indications of possible racial disparities in amyloid positivity risk and identified disparities in both ADI and risk factors for AD dementia. Black/African American and Latino participants showed lower rates of amyloid PET positivity compared to non-Latino White individuals, replicating our group’s previous results within a subset of the IDEAS cohort (*n* = 17,107)^26^ and concurring with results in non-Latino Black and White participants from the A4 study.^11^ Our findings in IDEAS contrast with studies in smaller cohorts that found either higher rates of amyloid PET positivity among Black/African American individuals^16^ or mixed amyloid differences between ethnoracial groups.^13–15,47–49^ Sample selection in limited geographic regions and from research cohorts not representative of clinical dementia populations could partially explain the mixed results across studies.

Beyond amyloid status, socioeconomic predictors and AD risk factors showed disparities across ethnoracial groups in IDEAS, reflecting the established body of work demonstrating the impact of social factors on health outcomes. Latino and Black/African American individuals had lower education attainment compared to non-Latino White participants, in concordance with previous research^26,50^ and Department of Education statistics^51^ showing 21.5% of Latino and 29% of Black individuals complete bachelor’s degrees compared to 42.3% of White individuals. The Black/African American group also had the greatest proportion of participants residing in highly disadvantaged neighborhoods, likely reflecting the disproportionate impact of housing discrimination on this population.^52^ We also found greater rates of hypertension among Black/African American individuals, compared to other ethnoracial groups;^53^ greater rates of diabetes among Black/African American, Latino, and Asian individuals;^54^ and greater levels of impairment at presentation among Black/African American, Latino, and Asian individuals,^45^ as reported widely across other biomedical studies.

### 4.3 | Strengths and limitations

This study benefits from a large ethnoracially diverse sample recruited from Medicare beneficiaries across the United States, which is representative of clinical populations receiving dementia care. Methodological strengths additionally include the use of a widely validated measure of neighborhood disadvantage, use of a large sample size of available data from the IDEAS cohort (though multiple imputation was used to account for missing data), and addressing the clustered structure of the IDEAS dataset in our analyses.

Our study also has important limitations. Because participation in the IDEAS study required enrollment through a dementia care specialist, patients from highly disadvantaged settings without access to specialized care are underrepresented in the sample. Proportionally low representation among Black/African American, Latino, and Asian participants compared to White individuals may have also limited our power to detect additional racial disparities. In IDEAS, racial and ethnic identity were provided via self-report but there were no instructions given to dementia specialists on how to ascertain this information in a standardized fashion. This has implications for our secondary analysis, as misclassification of individuals and other errors could have occurred in IDEAS given lack of a standardized procedure.

Missing ADI data and missing CL data are critical considerations as missing ADI data totaled ≈ 20% and missing CL data totaled > 40%. We used multiple imputation but also provided analyses examining only non-missing data. We did find differences between those with non-missing data compared to those with missing data (notably for ADI) and results should be interpreted in this context. We are largely limited in ability to correct for this given this is a secondary data analysis. Additionally, IDEAS did not capture fully comprehensive clinical or biomarker data. Only total MMSE and MoCA score were captured as objective cognitive measures. There were no data captured with further breakdown of these scores across domains, which limits our ability to further examine trends in cognitive profiles of participants. IDEAS did not capture MRI imaging data or other biomarker data to allow for in-depth evaluation of potential pathologies like cerebrovascular disease. There is also no longitudinal component of IDEAS that allows for autopsy data collection. The previously described mixed findings regarding racial differences in neuropathology cannot be further clarified by IDEAS given its design and this is an important area for future research. Apolipoprotein E genotypes were also unavailable, which could affect model estimates.

Historical residence data were unavailable; thus, we were unable to examine relationships between longitudinal neighborhood disadvantage exposure and amyloid positivity risk. Because amyloid begins accumulating ≈ 20 years before dementia onset,^55,56^ it is likely that high disadvantage earlier in life plays a role in predicting later-life amyloid burden and AD dementia risk. It is possible an individual can migrate between neighborhoods of varying disadvantage over the course of a lifetime, but prior work has demonstrated that ability to migrate into less disadvantaged neighborhoods differs between ethnoracial groups. Black/African American individuals are significantly less likely to migrate out of disadvantaged neighborhoods and more likely to migrate into disadvantaged neighborhoods.^57–59^ Long-lasting effects of residential segregation (as well as ongoing acts) significantly impact Black/African American individuals and limit where they can reside.

Though ADI is a composite measure, it emphasizes select domains and lacks measurement of variables like greenspace that are potentially important considerations. We also call attention to use of the 2022 v4 ADI, which contains data from the 2018 to 2022 American Community Survey. IDEAS concluded prior to this, which makes an earlier version of ADI potentially more appropriate for use. However, we were provided all ADI data from the IDEAS study team directly without ability to generate new ADI measures and thus we assume the 2022 v4 ADI version is comparable to a version that would have chronologically preceded IDEAS. Ongoing and future studies will need to capture a broader range of potentially influential factors to clarify the effect of residential neighborhood on amyloid status in dementia.

## 5 | CONCLUSION

Residence in the most disadvantaged neighborhoods is associated with lower rates of amyloid PET positivity, despite clinically significant cognitive impairment. Elucidating the role of amyloid and non-amyloid pathology in cognitive impairment among socioeconomically and ethnoracially diverse populations is of critical importance in seeking to better understanding the role of novel disease-modifying therapies in treatment for all individuals with AD and other causes of cognitive impairment. The results and outstanding questions from the current study highlight the need for future studies that (1) are larger in scope, (2) capture more cross-sectional and longitudinal social determinants of health data, (3) more deeply characterize biomarker status, (4) further stratify by ethnoracial identity and cognitive status to evaluate within-group effects, and (5) include aging adults from diverse ethnoracial and sociodemographic backgrounds.

## Supplementary Material

Supp

Supp1

Supp2

Supp3

Supp4

Supp5

Supp6

SUPPORTING INFORMATION

Additional supporting information can be found online in the Supporting Information section at the end of this article.

## Figures and Tables

**TABLE 1:** Participant characteristics by ethnoracial identity (*n* = 17,136).

	Total sample	White	Latino	Black/African American	Asian	*p* value*
No. (%)	17,136 (100)	15346 (89.6)	829 (4.8)	637 (3.7)	321 (1.9)	
Sex, no. (%)**						
Male	8352 (48.8)	7651 (49.9)	314 (37.9)	237 (37.2)	150 (46.7)	<0.001
Female	8781 (51.3)	7695 (50.1)	515 (62.1)	400 (62.8)	171 (53.3)	
Age, mean (SD) years	75.7 (6.3)	75.7 (6.3)	75.9 (6.7)	75.0 (6.5)	76.3 (6.3)	0.031
Education, no. (%)						
High school or less	5700 (33.3)	4749 (30.9)	521 (62.9)	327 (51.3)	103 (32.1)	<0.001
Some college or above	11,436 (66.7)	10,600 (69.1)	308 (37.2)	310 (48.7)	218 (67.9)	
English as primary language, no. (%)	16,266 (94.9)	15,099 (98.4)	363 (43.8)	629 (98.7)	175 (54.5)	<0.001
Medical comorbidities, no. (%)***						
Hypertension	8738 (51.0)	7705 (50.2)	449 (54.2)	434 (68.1)	150 (46.7)	<0.001
Other cardiovascular	8803 (51.4)	7937 (51.7)	408 (49.2)	304 (47.7)	154 (47.9)	0.295
Pulmonary	592 (3.5)	559 (3.6)	14 (1.7)	13 (2.0)	6 (1.9)	0.004
Diabetes	2759 (16.1)	2280 (14.9)	221 (26.7)	178 (27.9)	80 (24.9)	<0.001
Kidney disease	548 (3.2)	473 (3.1)	25 (3.0)	39 (6.1)	11 (3.4)	0.003
Mood disorder	3090 (18.0)	2843 (18.5)	152 (18.3)	55 (8.6)	40 (12.5)	<0.001
Cerebrovascular disease	2558 (14.9)	2291 (14.9)	123 (14.8)	98 (15.4)	46 (14.3)	0.975
Impairment level, no. (%)						
MCI	10,416 (60.8)	9571 (62.4)	370 (44.6)	306 (48.0)	169 (52.7)	<0.001
Dementia	6720 (39.2)	5778 (37.6)	459 (55.4)	331 (52.0)	152 (47.4)	
MMSE, mean (SD)	24.3 (5.1)	24.5 (4.9)	21.7 (5.9)	22.0 (5.8)	23.0 (5.5)	<0.001
Amyloid PET status, no. (%)						
Positive	10,532 (61.6)	9593 (62.6)	450 (54.3)	343 (54.0)	146 (45.5)	<0.001
National ADI decile, no. (%)						<0.001
1–10	2523 (18.8)	2337 (18.5)	120 (18.2)	47 (10.6)	119 (45.8)	
11–20	2267 (16.2)	2040 (16.2)	102 (15.5)	70 (15.8)	55 (21.2)	
21–30	2211 (15.8)	2024 (16.0)	109 (16.5)	52 (11.7)	26 (10.0)	
31–40	1933 (13.8)	1769 (14.0)	83 (12.6)	58 (13.1)	23 (8.9)	
41–50	1524 (10.9)	1390 (11.0)	72 (10.9)	47 (10.6)	15 (5.8)	
51–60	1091 (7.8)	977 (7.7)	56 (8.5)	50 (11.3)	8 (3.1)	
61–70	781 (5.6)	702 (5.6)	43 (6.5)	32 (7.2)	4 (1.5)	
71–80	618 (4.4)	559 (4.4)	29 (4.4)	28 (6.3)	2 (0.8)	
81–90	405 (2.9)	361 (2.9)	22 (3.3)	21 (4.7)	1 (0.4)	
91–100	535 (3.8)	466 (3.7)	24 (3.6)	38 (8.6)	7 (2.7)	

*Note:* Other cardiovascular conditions include congestive heart failure, atrial fibrillation, history of acute or myocardial infarction, ischemic heart disease, dyslipidemia; pulmonary conditions include COPD; kidney disease includes chronic kidney disease; mood disorder includes active depression, bipolar affective disorder, schizophrenia; cerebrovascular disease includes cerebrovascular disease without stroke, prior history of stroke, or transient ischemic attack.

Abbreviations: ADI, Area Deprivation Index; COPD, chronic obstructive pulmonary disease; MCI, mild cognitive impairment; MMSE, Mini-Mental State Examination; PET, positron emission tomography; SD, standard deviation.

* *p* values account for clustering of patient observations by clinic site.

** All percentages presented are based on non-missing responses.

*** Presence of each comorbidity was assessed separately so percentages do not add to 100.

**TABLE 2:** Participant characteristics by amyloid PET positivity rates (*n* = 18,256).

Variable	Positive PET	Negative PET	*p* value*
National ADI, no. (%)**			
1–90	8724 (61.4)	5495 (38.7)	<0.001
91–100	301 (53.6)	261 (46.4)	
Sex, no. (%)			
Male	5274 (59.4)	3611 (40.6)	<0.001
Female	5859 (62.6)	3507 (37.4)	
Age, years (mean [SD])	76.2 (6.3)	75.0 (6.3)	<0.001
Education, no. (%)			
High school or less	3748 (61.5)	2350 (38.5)	0.475
Some college or above	7389 (60.8)	4769 (39.2)	
Medical comorbidities, no. (%)***			
Hypertension			
Yes	5596 (60.1)	3723 (39.9)	0.016
No	5541 (62.0)	3396 (38.0)	
Other cardiovascular			
Yes	5712 (61.1)	3641 (38.9)	0.889
No	5425 (60.9)	3478 (39.1)	
Pulmonary			
Yes	312 (50.2)	309 (49.8)	
No	10,825 (61.4)	6810 (38.6)	<0.001
Diabetes			
Yes	1529 (51.6)	1435 (48.4)	
No	9608 (62.8)	5684 (37.2)	<0.001
Kidney disease			
Yes	317 (54.8)	262 (45.3)	
No	10,820 (61.2)	6857 (38.8)	0.006
Mood disorder			
Yes	1821 (55.8)	1444 (44.2)	
No	9316 (62.1)	5675 (37.9)	<0.001
Cerebrovascular disease		1159 (43.0)	
Yes	1536 (57.0)	5960 (38.3)	<0.001
No	9601 (61.7)		
Impairment level, no. (%)			
MCI	6108 (55.3)	4932 (44.7)	<0.001
Dementia	5029 (69.7)	2187 (30.3)	

*Note:* Other cardiovascular conditions include congestive heart failure, atrial fibrillation, history of acute or myocardial infarction, ischemic heart disease, dyslipidemia; pulmonary conditions include COPD; kidney disease includes chronic kidney disease; mood disorder includes active depression, bipolar affective disorder, schizophrenia; cerebrovascular disease includes cerebrovascular disease without stroke, prior history of stroke or transient ischemic attack.

Abbreviations: ADI, Area Deprivation Index; COPD, chronic obstructive pulmonary disease; MCI, mild cognitive impairment; PET, positron emission tomography; SD, standard deviation.

* *p* values account for clustering of patient observations by clinic site.

** Row and column number do not total 18,256 due to missing ADI data.

*** Presence of each comorbidity was assessed separately so percentages do not add to 100.

**TABLE 3:** Resultsfrom binomial logistic mixed effect model estimating the association of ADI and visual amyloid PET positivity (*n* = 18,293), all missing values imputed.

Variable	OR (95% CI)	*p* value
National ADI		
1–90	Ref	
91–100	0.80 (0.67, 0.96)	0.017
Age, years*		
Continuous	1.03 (1.02, 1.04)	<0.001
Sex*		
Male	Ref	
Female	1.16 (1.08, 1.26)	<0.001
Race/Ethnicity*		
White, non-Latino	Ref	
Latino	0.70 (0.57, 0.85)	<0.001
Black/African American	0.63 (0.53, 0.75)	<0.001
Asian	0.47 (0.35, 0.61)	<0.001
Education*		
High school or less	Ref	
Some college or above	1.09 (1.01, 1.17)	0.034
Primary language*		
English	Ref	
Spanish	0.87 (0.66, 1.16)	0.342
Other	0.84 (0.67, 1.06)	0.133
Medical comorbidities*		
Hypertension	0.93 (0.87, 0.98)	0.013
Other cardiovascular	1.06 (0.99, 1.14)	0.076
Pulmonary	0.63 (0.53, 0.74)	<0.001
Diabetes	0.67 (0.62, 0.74)	<0.001
Kidney disease	0.80 (0.67, 0.96)	0.015
Mood disorder	0.79 (0.72, 0.87)	<0.001
Cerebrovascular disease	0.77 (0.69, 0.85)	<0.001
Impairment level*		
MCI	Ref	
Dementia	1.24 (1.14, 1.36)	<0.001
MMSE score*		
Continuous	0.91 (0.90, 0.92)	<0.001

*Note:* Other cardiovascular conditions include congestive heart failure, atrial fibrillation, history of acute or myocardial infarction, ischemic heart disease, dyslipidemia; pulmonary conditions include COPD; kidney disease includes chronic kidney disease; mood disorder includes active depression, bipolar affective disorder, schizophrenia; cerebrovascular disease includes cerebrovascular disease without stroke, prior history of stroke or transient ischemic attack. None of results from omnibus tests for significance of interactions (ADI × ethnoracial identity; ADI × age; ADI × sex) were significant.

Abbreviations: ADI, Area Deprivation index; CI confidence interval; COPD, chronic obstructive pulmonary disease; MCI, mild cognitive impairment; MMSE, Mini-Mental State Examination; OR, odds ratio; PET, positron emission tomography.

* Each row item details the variables that were adjusted for in the model; effect estimates should not be assumed to be interpretable in a similar or identical manner to national ADI, the primary exposure.

**TABLE 4:** ADI effects on rates of quantitative amyloid PET positivity using multiple imputation (*n* = 18293).

Variable	CL ≥ 40 vs. CL ≤ 10 OR (95% CI)	*p* value	CL 11–39 vs. CL ≤ 10 OR (95% CI)	*p* value
National ADI				
1–90	Ref		Ref	
91–100	0.81 (0.66, 1.01)	0.060	0.74 (0.53, 1.02)	0.062
Age*				
Continuous	1.02 (1.02, 1.03)	<0.001	1.02 (1.01, 1.03)	<0.001
Sex*				
Male	Ref		Ref	
Female	1.28 (1.17, 1.40)	<0.001	1.15 (1.01, 1.31)	0.047
Race/Ethnicity*				
White, non-Latino	Ref		Ref	
Latino	0.73 (0.56, 0.99)	0.020	0.59 (0.38, 0.89)	0.013
Black/African American	0.80 (0.63, 1.01)	0.058	1.09 (0.79, 1.50)	0.614
Asian	0.53 (0.39, 0.73)	<0.001	0.67 (0.43, 1.06)	0.084
Education*				
High school or less	Ref		Ref	
Some college or above	1.13 (1.02, 1.25)	0.021	1.16 (1.01, 1.35)	0.047
Primary language*				
English	Ref		Ref	
Spanish	0.80 (0.58, 1.11)	0.174	1.17 (0.72, 1.89)	0.537
Other	0.76 (0.59, 1.06)	0.261	0.80 (0.54, 1.18)	0.110
Medical comorbidities*				
Hypertension	0.91 (0.84, 0.99)	0.040	0.91 (0.80, 1.03)	0.145
Other cardiovascular	1.13 (1.02, 1.24)	0.018	1.07 (0.94, 1.23)	0.294
Pulmonary	0.71 (0.56, 0.89)	0.003	0.74 (0.54, 0.99)	0.046
Diabetes	0.77 (0.69, 0.86)	<0.001	0.79 (0.68, 0.92)	0.002
Kidney disease	0.92 (0.73, 1.17)	0.495	0.99 (0.71, 1.36)	0.932
Mood disorder	0.82 (0.74, 0.92)	0.001	0.93 (0.81, 1.08)	0.359
Cerebrovascular disease	0.85 (0.75, 0.96)	0.011	0.99 (0.84, 1.16)	0.866
Impairment level*				
MCI	Ref		Ref	
Dementia	1.11 (0.99, 1.25)	0.084	1.01 (0.86, 1.18)	0.916
MMSE score*				
Continuous	0.94 (0.93, 0.95)	<0.001	0.95 (0.93, 0.97)	<0.001

*Note:* Other cardiovascular conditions include congestive heart failure, atrial fibrillation, history of acute or myocardial infarction, ischemic heart disease, dys- lipidemia; pulmonary conditions include COPD; kidney disease includes chronic kidney disease; mood disorder includes active depression, bipolar affective disorder, schizophrenia; cerebrovascular disease includes cerebrovascular disease without stroke, prior history of stroke or transient ischemic attack. None of results from omnibus tests for significance of interactions (ADI × ethnoracial identity; ADI × age; ADI × sex) were significant.

Abbreviations: ADI, Area Deprivation Index; CI, confidence interval; CL, Centiloid; COPD, chronic obstructive pulmonary disease; MCI, mild cognitive impairment; MMSE, Mini-Mental State Examination; OR, odds ratio; PET, positron emission tomography.

* Each row item details the variables that were adjusted for in the model; effect estimates should not be assumed to be interpretable in a similar or identical manner to national ADI, the primary exposure.

## RESEARCH IN CONTEXT

### 1. Systematic Review:

We reviewed the literature using traditional search engines (PubMed, Google Scholar) and found mixed findings regarding the impact of residence in a disadvantaged neighborhood on amyloid positron emission tomography (PET) positivity. Prior work also featured cohorts minimally inclusive of select ethnoracial groups.

### 2. Interpretation:

Residence in the most disadvantaged neighborhoods as defined by Area Deprivation Index (ADI) was associated with lower odds of amyloid PET positivity by visual interpretation but not quantitative measures. No ethnoracial identity × ADI interaction was found. Non-Alzheimer’s disease (AD) pathology may make important contributions to cognitive impairment among certain groups.

### 3. Future Directions:

Research investigating the complex relationship between the longitudinal impact of neighborhood residence, other social determinants of health, risk factors for AD, and cognitive impairment is needed. Inclusion of clinically heterogeneous, ethnoracially diverse patients is of critical importance in this future work.

## HIGHLIGHTS

- Residence in the most disadvantaged neighborhoods is associated with lower odds of amyloid positron emission tomography positivity.
- Ethnoracial identity and neighborhood residence interactions are complex.
- Non-amyloid pathology contributes to cognitive impairment among those residing in disadvantaged neighborhoods.
