# Supplementary material for: Association of residential neighborhood disadvantage with amyloid PET positivity among cognitively impaired individuals
Source: Alzheimers Dement Behav Socioecon Aging. Author manuscript; Available in PMC 2026 Mar 11. (PMC12973527; doi:10.1002/bsa3.70058)
Supplement: Supp1 [file NIHMS2146471-supplement-Supp1.docx]

**Supplemental Table 1. Geographic distribution of sample by race and ethnicity (n=17131) for individuals with non-missing data for race/ethnicity and region of residence**

|  | **Total Sample** | **White** | **Latino** | **Black/African American** | **Asian** |
| --- | --- | --- | --- | --- | --- |
| Region, no. (%) |  |  |  |  |  |
| Atlantic | 5991 (35.0) | 5299 (34.5) | 234 (28.2) | 352 (55.3) | 106 (33.0) |
| Central | 2465 (14.4) | 2323 (15.1) | 54 (6.5) | 68 (10.7) | 20 (6.2) |
| Southern | 5288 (30.9) | 4820 (31.4) | 289 (34.9) | 149 (23.4) | 30 (9.4) |
| Western-Pacific | 3387 (19.8) | 2902 (18.9) | 252 (30.4) | 68 (10.7) | 165 (51.4) |

*Regions geographically defined across the United States
