# Supplementary material for: Association of residential neighborhood disadvantage with amyloid PET positivity among cognitively impaired individuals
Source: Alzheimers Dement Behav Socioecon Aging. Author manuscript; Available in PMC 2026 Mar 11. (PMC12973527; doi:10.1002/bsa3.70058)
Supplement: Supp2 [file NIHMS2146471-supplement-Supp2.docx]

**Supplemental Table 2. Comparison of Individuals with Non-Missing ADI Data to Individuals with Missing ADI Data**

|  | **Non-missing data** | **Missing data** | **p-value** |
| --- | --- | --- | --- |
| Gender, N (%)  Male  Female  Other | 7345 (82.5)  7463 (79.6)  4 (80.0) | 1561 (17.5)  1919 (20.5)  1 (20.0) | <.001 |
| Race/ethnicity  White, non-Latino  Latino  Black/African American  Asian  Other  More than one race, non-Latino  Unknown race/ethnicity | 12625 (82.3)  660 (79.6)  443 (69.5)  260 (81.0)  25 (78.1)  24 (77.4)  775 (70.8) | 2724 (17.8)  169 (20.4)  194 (30.5)  61 (19.0)  7 (21.9)  7 (22.6)  319 (29.2) | <.001 |
| Education  High school or less  Some college or more | 4742 (77.6)  10070 (82.7) | 1370 (22.4)  2111 (17.3) | <.001 |
| Primary language  English  Spanish  Other | 14042 (81.1)  420 (78.2)  350 (78.5) | 3268 (18.9)  117 (21.8)  96 (21.5) | 0.095 |
| Hypertension  No  Yes | 7331 (81.9)  7481 (80.1) | 1621 (18.1)  1860 (19.9) | 0.002 |
| Other vascular comorbidities  No  Yes | 7287 (81.7)  7525 (80.3) | 1633 (18.3)  1848 (19.7) | 0.015 |
| Pulmonary comorbidities  No  Yes | 14296 (80.9)  516 (83.0) | 3375 (19.1)  106 (17.0) | 0.199 |
| Diabetes comorbidities  No  Yes | 12459 (81.3)  2353 (79.3) | 2866 (18.7)  615 (20.7) | 0.010 |
| Kidney comorbidities  No  Yes | 14356 (81.0)  456 (78.8) | 3358 (19.0)  123 (21.2) | 0.168 |
| Mood disorder comorbidities  No  Yes | 12098 (80.5)  2714 (83.1) | 2927 (19.5)  554 (17.0) | 0.001 |
| Cerebrovascular comorbidities  No  Yes | 12645 (81.1)  2167 (80.3) | 2949 (18.9)  532 (19.7) | 0.328 |
| Impairment type  MCI  Dementia | 9099 (82.3)  5713 (79.0) | 1961 (17.7)  1520 (21.0) | <.001 |
| MMSE score (range 0-30)  Mean ± SD | 24.3 ± 5.0 | 23.8 ± 5.2 | 0.007 |

MCI – mild cognitive impairment; MMSE – mini mental state examination
