# Supplementary material for: Association of residential neighborhood disadvantage with amyloid PET positivity among cognitively impaired individuals
Source: Alzheimers Dement Behav Socioecon Aging. Author manuscript; Available in PMC 2026 Mar 11. (PMC12973527; doi:10.1002/bsa3.70058)
Supplement: Supp3 [file NIHMS2146471-supplement-Supp3.docx]

**Supplemental Table 3. Results from Generalized Estimating Equation model with exchangeable correlation (listwise deletion model) estimating the association of ADI and visual amyloid PET positivity (n=13961)**

| **Variable** | **OR (95% CI)** | **p-value** |
| --- | --- | --- |
| National ADI    1-10  11-20  21-30  31-40  41-50  51-60  61-70  71-80  81-90  91-100 | Ref  1.00 (0.88, 1.14)  0.94 (0.83, 1.08)  0.92 (0.80, 1.06)  0.93 (0.80, 1.08)  0.94 (0.80, 1.11)  0.99 (0.83, 1.19)  0.99 (0.81, 1.21)  0.90 (0.71, 1.14)  0.78 (0.63, 0.96) | 0.995  0.388  0.261  0.330  0.488  0.928  0.935  0.370  0.017 |
| Age, years |  | <.001 |
| Continuous | 1.03 (1.02, 1.03) |  |
| Gender |  | <.001 |
| Male | Ref |  |
| Female | 1.16 (1.08, 1.26) |  |
| Race/Ethnicity |  | 0.002  <.001  <.001 |
| White, non-Latino | Ref |  |
| Latino | 0.69 (0.55, 0.87) |  |
| Black/African American | 0.61 (0.50, 0.75) |  |
| Asian | 0.45 (0.34, 0.60) |  |
| Education |  | 0.095 |
| High School or less | Ref |  |
| Some college or above | 1.07 (0.99, 1.17) |  |
| Primary Language |  | 0.147  0.782 |
| English | Ref |  |
| Spanish | 0.80 (0.59, 1.08) |  |
| Other | 0.96 (0.72, 1.28) |  |
| Medical Comorbidities |  | 0.075  0.173  <.001  <.001  0.003  <.001  <.001 |
| Hypertension | 0.93 (0.86, 1.01) |  |
| Other Cardiovascular | 1.06 (0.98, 1.14) |  |
| Pulmonary | 0.64 (0.53, 0.77) |  |
| Diabetes | 0.69 (0.63, 0.76) |  |
| Kidney Disease | 0.74 (0.60, 0.90) |  |
| Mood Disorder | 0.78 (0.71, 0.86) |  |
| Cerebrovascular Disease | 0.77 (0.69, 0.85) |  |
| Impairment Level |  | <.001 |
| MCI | Ref |  |
| Dementia | 1.20 (1.10, 1.32) |  |
| MMSE Score |  | <.001 |
| Continuous | 0.91 (0.90, 0.92) |  |

ADI – area deprivation index; Other Cardiovascular conditions include Congestive heart failure, atrial fibrillation, history of acute or myocardial infarction, ischemic heart disease, dyslipidemia; Pulmonary conditions include COPD; Kidney disease includes chronic kidney disease; Mood disorder includes active depression, bipolar affective disorder, schizophrenia; cerebrovascular disease includes cerebrovascular disease without stroke, prior history of stroke or TIA; MCI – mild cognitive impairment; MMSE – mini mental state examination
