# Supplementary material for: Association of residential neighborhood disadvantage with amyloid PET positivity among cognitively impaired individuals
Source: Alzheimers Dement Behav Socioecon Aging. Author manuscript; Available in PMC 2026 Mar 11. (PMC12973527; doi:10.1002/bsa3.70058)
Supplement: Supp4 [file NIHMS2146471-supplement-Supp4.docx]

**Supplemental Table 4. Comparison of Individuals with Non-Missing Centiloid Value Data to Individuals with Missing Centiloid Value Data Among Those with Non-missing ADI and Non-missing Amyloid PET Visual Interpretation Data**

|  | **Non-missing data** | **Missing data** | **p-value** |
| --- | --- | --- | --- |
| Gender, N (%)  Male  Female  Other | 5071 (69.2)  5199 (69.8)  4 (100.0) | 2257 (30.8)  2250 (30.2)  0 | 0.306 |
| Race/ethnicity  White, non-Latino  Latino  Black/African American  Asian  Other, non-Latino  More than one race, non-Latino  Unknown race/ethnicity | 8779 (69.7)  444 (67.3)  307 (69.5)  182 (70.0)  17 (68.0)  16 (66.7)  529 (68.9) | 3823 (30.3)  216 (32.7)  135 (30.5)  78 (30.0)  8 (32.0)  8 (33.3)  239 (31.1) | 0.921 |
| Education  High school or less  Some college or more | 3293 (69.6)  6981 (69.5) | 1438 (30.4)  3069 (30.5) | 0.861 |
| Primary language  English  Spanish  Other | 9732 (69.5)  293 (69.8)  249 (71.4) | 4280 (30.6)  127 (30.2)  100 (28.7) | 0.745 |
| Hypertension  No  Yes | 5156 (70.5)  5118 (68.6) | 2161 (29.5)  2346 (31.4) | 0.012 |
| Other vascular comorbidities  No  Yes | 5172 (71.1)  5102 (68.0) | 2102 (28.9)  2405 (32.0) | <0.001 |
| Pulmonary comorbidities  No  Yes | 9931 (69.6)  343 (66.6) | 4335 (30.4)  172 (33.4) | 0.145 |
| Diabetes comorbidities  No  Yes | 8637 (69.5)  1637 (69.6) | 3793 (30.5)  714 (30.4) | 0.889 |
| Kidney comorbidities  No  Yes | 9976 (69.6)  298 (65.4) | 4349 (30.4)  158 (34.7) | 0.050 |
| Mood disorder comorbidities  No  Yes | 8387 (69.5)  1887 (69.6) | 3683 (30.5)  824 (30.4) | 0.903 |
| Cerebrovascular comorbidities  No  Yes | 8795 (69.7)  1479 (68.4) | 3822 (30.3)  685 (31.7) | 0.204 |
| Impairment type  MCI  Dementia | 6443 (70.9)  3831 (67.2) | 2639 (29.1)  1868 (32.8) | <0.001 |
| MMSE score (range 0-30)  Mean ± SD | 24.4 ± 5.1 | 24.3 ± 5.0 | 0.227 |

MCI – mild cognitive impairment; MMSE – mini mental state examination
