# Supplementary material for: Association of residential neighborhood disadvantage with amyloid PET positivity among cognitively impaired individuals
Source: Alzheimers Dement Behav Socioecon Aging. Author manuscript; Available in PMC 2026 Mar 11. (PMC12973527; doi:10.1002/bsa3.70058)
Supplement: Supp5 [file NIHMS2146471-supplement-Supp5.docx]

**Supplemental Table 5. Results from Nominal Logistic Regression with cluster adjustment (listwise deletion model) estimating the association of ADI and Centiloid Value (N = 9717)**

|  | **CL 11-39 vs. CL≤10** | **p-value** | **CL≥40 vs. CL<10** | **p-value** |
| --- | --- | --- | --- | --- |
| **Variable** | **OR (95% CI)** |  | **OR (95% CI)** |  |
| National ADI  1-10  11-20  21-30  31-40  41-50  51-60  61-70  71-80  81-90  91-100 | --  1.09 (0.88, 1.34)  1.17 (0.95, 1.43)  1.14 (0.92, 1.41)  1.01 (0.80, 1.29)  1.19 (0.90, 1.56)  1.23 (0.89, 1.69)  1.57 (1.16, 2.13)  1.03 (0.69, 1.54)  0.82 (0.59, 1.14) | 0.433  0.149  0.218  0.916  0.227  0.214  0.003  0.891  0.240 | --  1.05 (0.90, 1.23)  1.11 (0.94, 1.31)  1.01 (0.82, 1.25)  1.03 (0.85, 1.24)  1.13 (0.91, 1.40)  1.11 (0.86, 1.45)  1.11 (0.86, 1.44)  1.03 (0.74, 1.42)  0.79 (0.61, 1.02) | 0.532  0.228  0.916  0.771  0.280  0.414  0.429  0.873  0.070 |
| Age, years  Continuous | 1.02 (1.01, 1.03) | 0.018 | 1.03 (1.02, 1.04) | <.001 |
| Gender  Male  Female | Ref  1.09 (0.95, 1.27) | 0.223 | Ref  1.42 (1.27, 1.59) | <.001 |
| Race/ethnicity  White, non-Latino  Latino  Black/African American  Asian | --  0.65 (0.41, 1.04)  1.22 (0.90, 1.67)  0.77 (0.48, 1.24) | 0.073  0.205  0.291 | --  0.66 (0.49, 0.90)  0.63 (0.49, 0.82)  0.37 (0.25, 0.56) | 0.008  <0.001  <0.001 |
| Education  High school or less  Some college or more | Ref  1.15 (0.98, 1.38) | 0.088 | Ref  1.22 (1.08, 1.38) | 0.002 |
| Primary language  English  Spanish  Other | --  1.12 (0.64, 1.96)  0.88 (0.59, 1.31) | 0.699  0.516 | --  0.57 (0.38, 0.84)  0.71 (0.51, 0.99) | 0.005  0.047 |
| Medical Comorbidities |  | 0.302  0.651  0.264  0.011  0.587  0.506  0.890 |  | 0.013  0.001  <.001  <.001  0.187  <.001  0.001 |
| Hypertension | 0.94 (0.82, 1.06) |  | 0.88 (0.79, 0.97) |  |
| Other vascular comorbidities | 1.03 (0.89, 1.20) |  | 1.20 (1.07, 1.34) |  |
| Pulmonary comorbidities | 0.85 (0.64, 1.13) |  | 0.60 (0.47, 0.76) |  |
| Diabetes comorbidities | 0.80 (0.68, 0.95) |  | 0.70 (0.62, 0.80) |  |
| Kidney comorbidities | 0.91 (0.65, 1.28) |  | 0.82 (0.61, 1.10) |  |
| Mood disorder comorbidities | 0.95 (0.82, 1.10) |  | 0.74 (0.64, 0.84) |  |
| Cerebrovascular comorbidities | 1.01 (0.86, 1.20) |  | 0.79 (0.68, 0.91) |  |
| Impairment type  MCI  Dementia | Ref  0.97 (0.82, 1.15) | 0.735 | Ref  1.15 (1.01, 1.32) | 0.039 |
| MMSE score  Continuous | 0.96 (0.94, 0.98) | <.001 | 0.90 (0.89, 0.92) | <.001 |

ADI – area deprivation index; Other Cardiovascular conditions include Congestive heart failure, atrial fibrillation, history of acute or myocardial infarction, ischemic heart disease, dyslipidemia; Pulmonary conditions include COPD; Kidney disease includes chronic kidney disease; Mood disorder includes active depression, bipolar affective disorder, schizophrenia; cerebrovascular disease includes cerebrovascular disease without stroke, prior history of stroke or TIA; MCI – mild cognitive impairment; MMSE – mini mental state examination
