# Supplementary material for: Association of residential neighborhood disadvantage with amyloid PET positivity among cognitively impaired individuals
Source: Alzheimers Dement Behav Socioecon Aging. Author manuscript; Available in PMC 2026 Mar 11. (PMC12973527; doi:10.1002/bsa3.70058)
Supplement: Supp6 [file NIHMS2146471-supplement-Supp6.docx]

**Supplemental Figure 1. Total number of IDEAS participants included in each Table or Figure**

**Total Participants in IDEAS (n=18,293)**

**Table 1**

**Included:** **17,136** with non-Latino White, Latino, Black/African American or Asian race/ethnicity

**Excluded: 1,157** with “other/prefer not to answer”

race/ethnicity

**Table 2**

**Included:** **18,256** with non-missing data on visual Amyloid PET

**Excluded: 37** with missing data on visual Amyloid PET

**Table 3 and 4**

**Included: 18,293**

Values were imputed for ALL missing observations
